# Climatic change controls productivity variation in global grasslands

**DOI:** 10.1038/srep26958

**Published:** 2016-05-31

**Authors:** Qingzhu Gao, Wenquan Zhu, Mark W. Schwartz, Hasbagan Ganjurjav, Yunfan Wan, Xiaobo Qin, Xin Ma, Matthew A. Williamson, Yue Li

**Affiliations:** 1 Institute of Environment and Sustainable Development in Agriculture, Chinese Academy of Agricultural Sciences, Beijing 100081, China; 2 Key Laboratory for Agro-Environment & Climate Change, Ministry of Agriculture, Beijing 100081, China; 3 College of Resources Science and Technology, Beijing Normal University, Beijing 100875, China; 4 John Muir Institute of the Environment, University of California, Davis, CA 95616, USA

## Abstract

Detection and identification of the impacts of climate change on ecosystems have been core issues in climate change research in recent years. In this study, we compared average annual values of the normalized difference vegetation index (NDVI) with theoretical net primary productivity (NPP) values based on temperature and precipitation to determine the effect of historic climate change on global grassland productivity from 1982 to 2011. Comparison of trends in actual productivity (NDVI) with climate-induced potential productivity showed that the trends in average productivity in nearly 40% of global grassland areas have been significantly affected by climate change. The contribution of climate change to variability in grassland productivity was 15.2–71.2% during 1982–2011. Climate change contributed significantly to long-term trends in grassland productivity mainly in North America, central Eurasia, central Africa, and Oceania; these regions will be more sensitive to future climate change impacts. The impacts of climate change on variability in grassland productivity were greater in the Western Hemisphere than the Eastern Hemisphere. Confirmation of the observed trends requires long-term controlled experiments and multi-model ensembles to reduce uncertainties and explain mechanisms.

Primary production and its trends are important indicators of ecosystem function^1^. Many studies have documented that ecosystem primary production is sensitive to climate change, and is also simultaneously responsive to many other non-climate factors in the world^2^,^3^,^4^. Since the Industrial Revolution, global terrestrial ecosystem net primary productivity has increased by approximately 5% compared with that in the preindustrial period^2^,^3^,^4^. However, because of the occurrence of multiple drivers, at any given location the ecosystem net primary productivity may have increased, not changed, or decreased, and responded in non-additive ways^4^. Grasslands are defined as areas where the vegetation is dominated by grasses. These areas are located mainly between forests and deserts, and between forests and ice-covered regions, and occupy approximately 30% of the earth’s ice-free land surface (Supplementary Fig. S1)^5^,^6^. Grassland productivity is fundamental to carbon sequestration and food chains on which humans and many herbivores depend^5^,^6^,^7^. Changes in primary productivity have shown varying patterns among global grassland ecosystems, and the nature and causes of this variability are debated^1^,^8^,^9^,^10^. Variability in the productivity of dry grasslands is significantly correlated with precipitation changes^11^,^12^,^13^. Grassland primary production has increased with warming in cold regions, but has decreased in hot regions^4^,^14^,^15^,^16^. In general, grassland primary production is extremely sensitive to precipitation and temperature changes, and to non-climate factors including grazing, fires, nitrogen deposition, and rising CO_2_ levels^4^,^17^. Uncertainty in the response of grassland primary production to climate change and other perturbations remains a major impediment to assessing causal relationships, and to determining the levels of permissible climate change^4^. The gaps in knowledge have led to questions about how to detect and identify the impacts of climate change on the variability of grassland productivity at the global scale, relative to the effects of non-climate factors.

In this study, we identified the relative impacts of climate change on global grassland ecosystem productivity. We used the annual mean normalized difference vegetation index (NDVI) as a proxy for the actual primary productivity of the global grassland ecosystem, which reflects the effects of climatic, anthropogenic, and edaphic factors. The NDVI has been widely used in studies of a variety of ecosystem parameters, including vegetation biomass, activity, and phenological dynamics at regional and global scales^18^,^19^,^20^,^21^,^22^,^23^. We then applied the climate-driven Miami Model to simulate the potential net primary production (NPP) of the global grassland ecosystem; this model estimates NPP strictly as a function of temperature and precipitation. This process enabled us to identify the contribution of climate to NPP, and demonstrated the integrated and overlapping impacts of climate factors on productivity change. We used centered, standardized estimates (standardized anomalies; SAs) of NDVI and NPP to assess trends in productivity. To identify major changes, we calculated the coefficient of determination to assess the degree to which changes in NPP were correlated with changes in the NDVI.

## Results

### Spatial trends in grassland productivity

**Figure 1:**
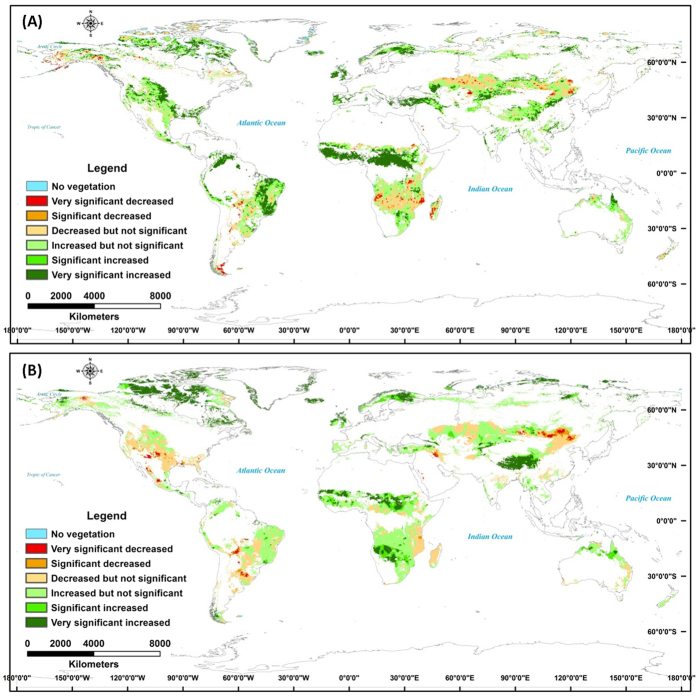
Spatial trends of annual mean NDVI (**A**) and potential NPP (**B**) of global grassland from 1982 to 2011. The spatial maps of annual mean NDVI and potential NPP trends in global grassland ecosystems were developed from the spatial correlation technique through the application of ERDAS IMAGINE 8.4 (http://www.hexagongeospatial.com/products/producer-suite/erdas-imagine) and ArcGIS 10 (http://www.esri.com/software/arcgis/arcgis-for-desktop).

Approximately 60% of global grasslands showed no significant variation in the mean annual NDVI during 1982–2011 (Fig. 1A). The mean NDVI value increased significantly in 36.3% of global grassland areas, and decreased significantly in 4.6%. Trends of significant increase occurred in mid-eastern South America, central Africa, central Eurasia, the high-latitude regions, and the Qinghai–Tibetan Plateau. Trends of significant decrease occurred primarily on the Mongolian Plateau and in central Eurasia (Fig. 1A).

Model-derived potential NPP values showed larger trends of decrease and smaller trends of increase than did the values for the annual mean NDVI. The largest trends of decrease in the model-driven NPP values were found for the Mongolian Plateau, the Midwestern USA, and in mid-eastern South America, while the largest trends of increase were found for the Qinghai–Tibetan Plateau, the high-latitude regions, and central Africa (Fig. 1B). This comparison indicates that actual changes in grassland primary productivity (as assessed by the NDVI) were more widespread than would be predicted by variability in climate alone.

### Different trends in potential and actual productivity in grassland ecosystems

**Figure 2:**
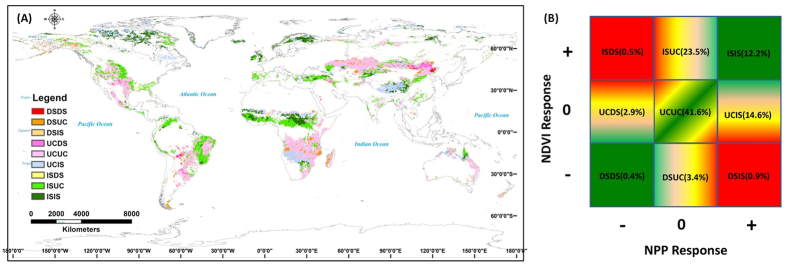
Spatial distribution (**A**) and proportion (**B**) of different trend regions in global grassland ecosystem. The DSDS is the region of annual mean NDVI decreased significantly and potential NPP decreased significantly, DSUC is the region of annual mean NDVI decreased significantly and potential NPP unchanged significantly, DSIS is the region of annual mean NDVI decreased significantly and potential NPP increased significantly, UCDS is the region of annual mean NDVI unchanged significantly and potential NPP decreased significantly, UCUC is the region of annual mean NDVI unchanged significantly and potential NPP unchanged significantly, UCIS is the region of annual mean NDVI unchanged significantly and potential NPP increased significantly, ISDS is the region of annual mean NDVI increased significantly and potential NPP decreased significantly, ISUC is the region of annual mean NDVI increased significantly and potential NPP unchanged significantly, ISIS is the region of annual mean NDVI increased significantly and potential NPP increased significantly. “+” means increased, “0” is unchanged and “−” is decreased, the data in parenthesis of figure b are the proportion of different trend regions. The spatial map of different trend regions in global grassland ecosystems was developed from the spatial overlap technique through the application of ArcGIS 10 (http://www.esri.com/software/arcgis/arcgis-for-desktop).

For the period 1982–2011, the actual productivity (annual mean NDVI) and model-derived potential NPP showed different trends with respect to the global grassland ecosystem. We overlaid the trends in actual and potential productivity to distinguish nine trend regions (see Methods) for identifying the impacts of climate change on variation in global grassland productivity (Fig. 2). Among the nine trend regions the grasslands had a similar (both the annual mean NDVI and the model-derived NPP decreased significantly (DSDS) or increased significantly (ISIS) regions) or no obvious (neither the annual mean NDVI nor the model-derived NPP showed a significant change (UCUC) region) trend in actual and potential productivity. These similar or no obvious changed regions comprise more than 50% of the global grassland area. The grassland regions showing opposite trends (the annual mean NDVI decreased significantly and the model-derived NPP increased significantly (DSIS), and the annual mean NDVI increased significantly and the model-derived NPP decreased significantly (ISDS)) constituted a very small proportion (<1.5%) of the total global grassland area. Among the four largest regions (UCUC, UCIS (the annual mean NDVI changed insignificantly and the model-derived NPP increased significantly region), ISUC (the annual mean NDVI increased significantly and the model-derived NPP changed insignificantly region), and ISIS), the UCUC region comprises grassland areas mainly outside of the high-latitude region. Climate-related potential productivity increased significantly during the study period, but inconsistent changes in the NDVI values were found for the region of lowest productivity (UCIS), which mainly occurs on the Qinghai–Tibetan Plateau, in central Africa, and in the high-latitude regions (Fig. 2 and Table 1). Actual productivity increased and climatic potential productivity remained unchanged in the ISUC region, which is mainly distributed in mid-eastern South America, central Africa, central Eurasia, and the Midwestern USA. Both actual and climatic potential productivity increased significantly in a single low-productivity region (ISIS), which is mainly distributed in central Africa, the high-latitude regions, Oceania, and the Qinghai–Tibetan Plateau (Fig. 2 and Table 1). The abrupt points in the trend of decrease in the annual mean NDVI occurred during the period 2000–2004, whereas abrupt points in the trend of increase in the annual mean NDVI occurred during the period 1995–2000 (Table 1). The abrupt points in the potential NPP in the period 1995–2001 usually occurred earlier than those in the NDVI (Table 1).

### Climate change contributions to variability in grassland productivity

**Figure 3:**
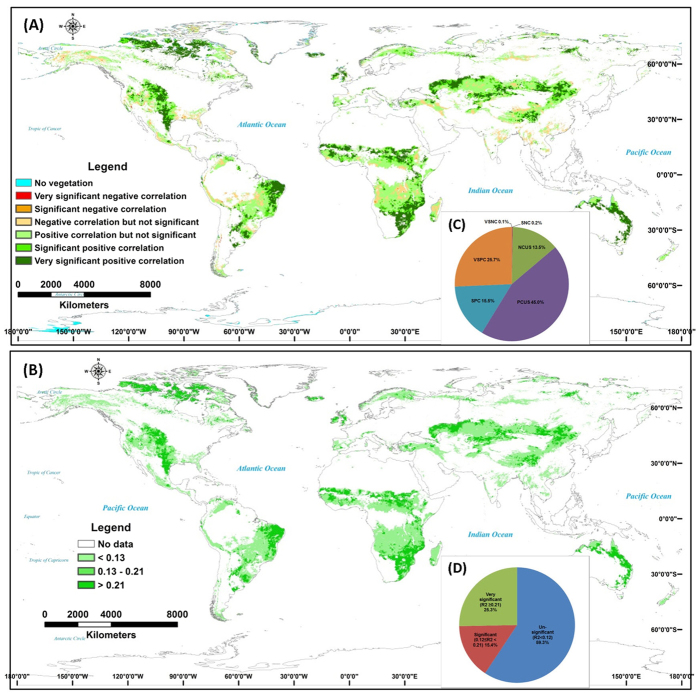
Spatial correlations (**A**) and its proportions (**C**) and coefficient of determination (**B**) and its proportions (**D**) of annual mean NDVI and potential NPP in global grassland ecosystem. The VSNC is very significant negative correlation, SNC is significant negative correlation, NCNS is negative correlated but not significantly, PCNS is positive correlated but not significantly, SPC is significant positive correlation, VSPC is very significant positive correlation. The spatial correlation maps between annual mean NDVI and potential NPP in global grassland ecosystem were developed from the spatial correlation technique through the application of ERDAS IMAGINE 8.4 (http://www.hexagongeospatial.com/products/producer-suite/erdas-imagine) and ArcGIS 10 (http://www.esri.com/software/arcgis/arcgis-for-desktop).

During the period 1982–2011, for 58.5% of the global grassland area there was no significant (p > 0.05) correlation between the annual mean NDVI and the potential NPP (Fig. 3C). Areas having significant or highly significant negative correlations between the annual mean NDVI and the potential NPP accounted for 0.3% of the global grassland area, whereas 15.5% and 25.7% of the area showed significant positive and highly significant positive correlations, respectively. These correlations were mainly distributed in the high-latitude regions, central Eurasia, Midwestern USA, the Mongolian Plateau, central Africa, and Oceania (Fig. 3A).

Based on the coefficient of determination between the SAs for the annual mean NDVI and the potential NPP, for regions showing significant correlations between the variables, the contribution of climate change to variability in grassland productivity ranged from 15.2% to 71.2% (Table 2). The mean contribution of climate change to variability in grassland productivity in the ISIS region reached 71.2% (Table 2). The contributions of climate change were significant (R^2^ > 0.13, p < 0.05) and highly significant (R^2^ > 0.21, p < 0.01) for 15.4% and 25.3% of the total grassland area, respectively (Fig. 3D). These regions, which are mainly distributed in the high-latitude regions, Midwestern USA, the Mongolian Plateau, central Eurasia, central Africa, and Oceania (Fig. 3B), will be particularly sensitive to future climate change.

### Spatial patterns in the contribution of climate change to variability in grassland productivity

**Figure 4:**
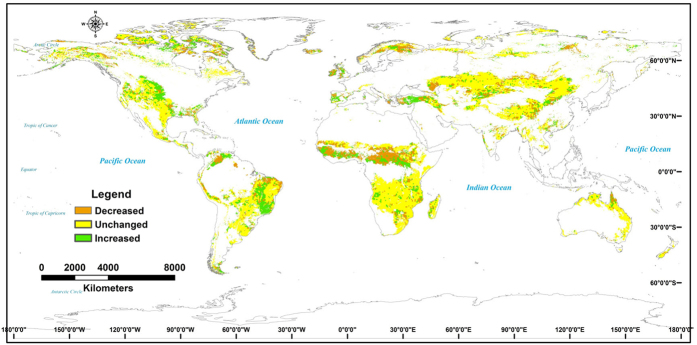
The changes of determination coefficient (*R*^*2*^) of correlation between annual mean NDVI and potential NPP in global grasslands after 2000. The spatial change maps were developed from the spatial correlation and calculation techniques through the application of ERDAS IMAGINE 8.4 (http://www.hexagongeospatial.com/products/producer-suite/erdas-imagine) and ARCGIS 10 (http://www.esri.com/software/arcgis/arcgis-for-desktop).

The coefficient of determination for the correlation between the annual mean NDVI and the potential NPP decreased following abrupt points in the annual mean NDVI for the ISDS and ISIS regions, whereas in other regions it increased (Fig. 4). The areas in which the contribution of climate change to variability in productivity increased were mainly in the high-latitude regions, Midwestern USA, central Africa, and mid-eastern South America, whereas the areas where the contribution of climate change decreased were in central Eurasia, the Qinghai–Tibetan Plateau, and Oceania (Fig. 4). Thus, greater variation in the contribution of climate change to productivity occurred in central Africa and mid-eastern South America than in other areas.

## Discussion

### Detection and attribution of climate change impacts on global grassland productivity

The detection and attribution of climate change impacts on ecosystems, based on the relative contribution of climate change to the observed ecosystem changes, are core issues in climate change research, as discussed in the fifth assessment report (AR5) of the Intergovernmental Panel on Climate Change (IPCC)^24^. Tremendous progress has been made in recent years in detecting changes in regional terrestrial ecosystems, based on long-term observational and remote sensing series data^25^,^26^,^27^,^28^,^29^. However, distinguishing the effects on ecosystems of changes in temperature and precipitation from those of other anthropogenic change remains a complex and challenging task^30^. In this study we used a simple linear regression method to analyze the trends in actual and climate-driven grassland productivity, and used the Mann–Kendall (MK) test to detect abrupt points in the variability of grassland productivity in the different regions. Our results showed that grassland productivity remained unchanged or increased, depending on the region, and confirm the results of previous studies documenting vegetation changes in recent years^25^,^31^. However, the trends of grassland productivity in different regions may not be consistent with the results of previous researches due to the inconsistency of time periods^23^. Xia *et al.*^23^ used the period of 1982–2006 whereas we detected the grassland productivity changes from 1982–2011 in this study. Among grassland regions, the abrupt points in the trends in potential NPP usually occurred earlier than those in the NDVI (Table 1), indicating that changes in climate conditions generally precede ecosystem change^24^. This is in accordance with results previously reported by Wu *et al.*^32^ and Zhang *et al.*^32^ who found that climate change has a relatively slow effect on grassland productivity due to the vegetation adaptation^32^,^33^. The time-lag phenomenon is very important for accurately revealing the response of ecosystem productivity^32^, and would be one of key implications for adapting to future climate change impacts on global grassland ecosystem. Correlations between climate change impacts and detected ecosystem changes have predominantly been based on analyses of long-term series of spatial patterns using regional-scale remote sensing data^34^,^35^,^36^, as well as comparisons of remote sensing data with the results of ecosystem model simulations that control for driving factors^37^. In this study we combined remote sensing monitoring with climate-driven model simulation, and obtained estimates of the coefficient of determination for SAs for actual and potential productivity, which enabled the integrated impacts of climate change on changes in grassland productivity to be distinguished. Our results show that the contribution of climate change to variability in grassland productivity in significantly affected areas (approximately 40% of the total area) was 15–71% (Table 2). Based on a spatial pattern analysis alone, the contribution of climate change to global grassland change during the period 1982–2011 would have been only 40% (i.e., 15% significant change and 25% highly significant change) (Fig. 3D).

### Regional differences between actual and potential productivity change in global grasslands

The variability in actual and potential productivity varied throughout the global grassland ecosystem, and showed various trends in different grassland regions (Fig. 1 and Supplementary Fig. S2). The potential NPP increased significantly because of climate change during the study period, but human activities may have driven the unsynchronized changes in the NDVI values in some regions. These trends in grassland productivity may have been affected by overgrazing on the Qinghai–Tibetan Plateau^38^, and other non-climate factors including fires and shrub encroachment in mid-eastern South America and central Africa^39^,^40^. The significant positive correlations between actual and potential productivity were found mainly in the high-latitude regions, central Eurasia, Midwestern USA, the Mongolian Plateau, and Oceania (Fig. 3). These observations confirmed the accelerating effects of global warming on increased ecosystem productivity in the Arctic tundra^41^. The impacts of climate change dominated variability in the productivity of the grasslands of the Mongolian Plateau (Fig. 3). This finding differed from those of other studies that cited overgrazing was the key factor driving decreased grassland productivity in the northern part (Mongolia) of the Mongolian Plateau^19^,^20^. In general, overgrazing has the negative impacts on grassland ecosystems, while other human activities, including ecological protection and the establishment of nature reserves, have had positive ecological effects on grassland ecosystem productivity^22^,^42^. The prairies of the Midwestern USA and grasslands in Oceania were generally under less grazing pressure^5^, and the variability in their productivity has been mainly a consequence of climate change. However, more complex interactions between the impacts of climate change and non-climate factors (grazing, fires, woody encroachment, and other human activities) have governed the variability in global grassland productivity.

### Spatial variations in the impacts of climate change on global grassland productivity

Understanding the impacts of climate change is important in assessing the adaptation strategies of grasslands under conditions of future climate change, and change in the spatial and temporal patterns of terrestrial ecosystems^24^. A lesser global warming effect on vegetation activity in the Northern Hemisphere was apparent in the partial correlation between the NDVI and temperature^43^. We found that climate change has reduced the contribution to variability in productivity in Eurasian grasslands, but increased the contribution to variability in North America. Thus, no lessening of the effects of climate change on grassland productivity was observed in the Northern Hemisphere (Fig. 4). Most of the areas where the impact of climate change has increased the variability in productivity, including the high-latitude regions, Midwestern USA, central Africa, and mid-eastern South America, are in the Western Hemisphere, whereas in the Eastern Hemisphere there has been a reduction in impact on most of the grassland areas, including in central Eurasia, the Qinghai–Tibetan Plateau, and Oceania (Fig. 4). Thus, the contribution of climate change to variability in grassland productivity has been very different in the Western and Eastern hemispheres.

### Limitations of this study

Although we have provided measures of the relative contributions of climate change to variability in global grassland productivity, based on long-term time series data for the NDVI and model-derived potential NPP, the data are subject to uncertainty^21^,^32^. The NDVI has been widely used as a satellite proxy for ecosystem productivity, but it remains questionable whether it is comparable to productivity^44^. The GIMMS3g NDVI time series data also showed an inconsistency between sensors in different regions^45^. The model-derived potential NPP values, which we estimated using the Miami Model based on precipitation and temperature data, are also affected by other climate factors (e.g. solar radiation)^46^. However, the ambient CO_2_ concentration which has been reported by IPCC report was considered as one of important impact factors for the variations of primary production in terrestrial ecosystem in recent years^24^,^47^. The land cover map is also associated with uncertainty in this study, because the IGBP global land cover data also have some problems in classification of land cover types^48^ and land use should be having some changes in the grassland regions from 1982 to 2011. Future studies should refine the model by focusing on these uncertainties. In addition, the mechanisms underlying the relative contribution of climate change to the variability in global grassland productivity should be investigated using long-term controlled experiments and multi-model ensembles.

## Methods

### Study area

Grasslands comprise one of the largest ecosystems worldwide, and contribute to the livelihood of more than 800 million people^5^. They provide food, energy, wildlife foraging areas and habitats, carbon and water storage, and watershed protection for many major river systems^5^,^6^,^7^,^23^. Based on land cover data and classes defined by the International Geosphere–Biosphere Programme (IGBP, http://www.igbp.net), the area of global grasslands, which includes savanna and temperate grasslands, is 3.73 billion ha, representing about 19% of the world’s land area. Grasslands are mainly distributed in the high-latitude regions (tundra), the Midwestern USA, mid-eastern South America, central Africa, central Eurasia, the Mongolian Plateau, the Qinghai–Tibetan Plateau, and Oceania (Supplementary Fig. 1).

### Climate data

Monthly mean temperature and total precipitation data were obtained from the Climate Research Unit (CRU) Time-Series version 3.21 (TS3.21) of the High Resolution Gridded Data of Month-by-month Variation in Climate dataset (http://badc.nerc.ac.uk), at a resolution of 0.5° (Supplementary Table S1). These data were used to simulate the grassland potential productivity and to identify the impacts of climate change on the dynamics of global grassland productivity during 1982–2011.

### Grassland productivity data

We used the Advanced Very High Resolution Radiometer third-generation NDVI dataset developed by the Global Inventory Modeling and Mapping Studies (GIMMS) group (http://ecocast.arc.nasa.gov/data/pub/gimms/). The monthly GIMMS3 g NDVI data for 1982–2011, at a resolution of 0.083°, were processed to analyze the actual status of global grassland ecosystem productivity. The GIMMS3 g NDVI data have been calibrated and widely used to detect changes in vegetation at regional and global scales^23^,^45^,^49^,^50^.

### Grassland distribution

We used global land cover data from IGBP (http://www.igbp.net), at a resolution of 8000 m, to derive the global distribution of grasslands (including temperate grasslands and savanna in 17 IGBP classification types)^51^.

### Data processing

The gridded annual total precipitation and mean temperature were calculated by processing the monthly CRU TS3.21 data for the period 1982–2011. The annual mean values of the NDVI, obtained from the monthly GIMMS3 g NDVI for the period 1982–2011, were used to represent actual grassland productivity. The NDVI and grassland distribution map were resampled using the nearest-neighbor method at 0.5° resolution, to ensure the data resolution was appropriate for analysis of the impacts of climate change on variations in productivity in grassland ecosystems.

### Model simulations

We used the Miami Model to simulate the potential NPP of the global grassland ecosystem. This model has been widely used to calculate the climate-driven potential NPP for large areas and at the global scale^52^,^53^. We then compared the trends in potential NPP with those of the NDVI to identify the impacts of climate change on changes in global grassland productivity. The Miami Model simulated the potential NPP from the annual mean temperature (*T*, °C) and precipitation (*R*, mm) using the following equations^54^:


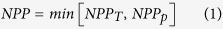



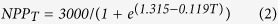



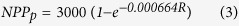


where *NPP* is the potential net primary productivity (gDM m^−2^ yr^−1^); *NPP*_*T*_ and *NPP*_*p*_ are the potential *NPP* of temperature and precipitation, respectively; T is the annual mean temperature (°C); and R is the annual precipitation (mm).

### Detection of general trends

We detected a gradual change in the actual productivity (mean NDVI) and potential NPP in grassland ecosystems for the period 1982–2011 at each pixel, in mainly distribution regions. Trends were detected using the linear model y = a + bx^21^,^55^, where a and b are regression coefficients (a is the intercept and b is the trend slope), y is the annual mean NDVI or model-derived potential NPP year by year, and x is time. The trends were identified as being statistically significant (p < 0.05) or highly significant (p < 0.01).

### Analysis of regional trend variations

To detect and identify changes in grassland productivity we overlaid the trends in the actual (annual mean NDVI) and model-derived productivity (potential NPP), and distinguished nine different trend regions with the following characteristics: (1) the annual mean NDVI and the model-derived NPP both decreased significantly (DSDS) region; (2) the annual mean NDVI decreased significantly and the model-derived NPP showed a non-significant change (DSUC) region; (3) the annual mean NDVI decreased significantly and the model-derived NPP increased significantly (DSIS) region; (4) the annual mean NDVI showed no significant change and the model-derived NPP decreased significantly (UCDS) region; (5) neither the annual mean NDVI nor the model-derived NPP showed a significant change (UCUC) region; (6) the annual mean NDVI changed insignificantly and the model-derived NPP increased significantly (UCIS) region; (7) the annual mean NDVI increased significantly and the model-derived NPP decreased significantly (ISDS) region; (8) the annual mean NDVI increased significantly and the model-derived NPP changed insignificantly (ISUC) region; and (9) both the annual mean NDVI and the model-derived NPP increased significantly (ISIS) region.

### Detection of abrupt points

We used the nonparametric Mann–Kendall (MK) test to estimate the abrupt points for all pixel values for the mean NDVI and potential NPP, aggregated over time for the various trend regions. The MK test is a useful exploratory method for identifying monotonic changes during specific time intervals, and has been widely used to test for trends in NDVI series data^35^.

### Spatial correlation

To identify the effects of climate change on global grassland productivity we calculated Pearson correlation coefficients (*r* values) to assess the relationships between mean NDVI and potential NPP values for each grid cell over the period 1982–2011.

### Attribution of climate change impacts

To compare the variability in the actual productivity (assessed using the NDVI) and model-derived NPP values (based on temperature and precipitation), we first estimated the standardized anomalies (SAs) for the aggregated time series of pixel values for the NDVI and potential NPP in the various trend regions (Supplementary Fig. S2). The SAs, which are also referred to as normalized anomalies, are calculated by dividing the anomalies by the standard deviation. The SAs generally provide more information about the magnitude of the anomalies, because the influences of dispersion have been removed.

To identify climate change impacts on grassland productivity, we then calculated the coefficient of determination (R^2^) between the standardized anomalies of the annual mean NDVI and potential NPP values for the various trend regions. To analyze changes in the contribution of climate change factors to the variability in grassland productivity in the various trend regions, and at the global scale, we compared the coefficients of determination for the SAs for the annual mean NDVI and the potential NPP values, before and after the abrupt points in the annual mean NDVI. The coefficient of determination indicated the contribution of climate factors to grassland productivity^12^,^56^.

## Additional Information

**How to cite this article**: Gao, Q. *et al.* Climatic change controls productivity variation in global grasslands. *Sci. Rep.* **6**, 26958; doi: 10.1038/srep26958 (2016).

## Supplementary Material

Supplementary Information

## Figures and Tables

**Table 1:** Distribution area, annual mean NDVI and its Mann-Kendall test in different trend regions of global grassland ecosystem.

Different trend regions	Area percentage (%)	Mean value ± SD of annual mean NDVI	MK-stat		Abrupt points	
			NDVI	NPP	NDVI	NPP
1. NDVI decreased significantly and NPP decreased significantly (DSDS)	0.4	0.424 ± 0.012	−4.37	−4.48	2000	1998
2. NDVI decreased significantly and NPP unchanged significantly (DSUC)	3.4	0.432 ± 0.010	−4.69	−0.55	2004	–
3. NDVI decreased significantly and NPP increased significantly (DSIS)	0.9	0.323 ± 0.009	−4.05	4.80	2001	1997
4. NDVI unchanged significantly and NPP decreased significantly (UCDS)	2.9	0.412 ± 0.006	0.73	−5.05	–	2001
5. NDVI unchanged significantly and NPP unchanged significantly (UCUC)	41.6	0.458 ± 0.005	1.02	1.02	–	–
6. NDVI unchanged significantly and NPP increased significantly (UCIS)	14.6	0.274 ± 0.006	1.23	5.23	–	1997
7. NDVI increased significantly and NPP decreased significantly (ISDS)	0.5	0.526 ± 0.013	4.62	−4.51	2000	1996
8. NDVI increased significantly and NPP unchanged significantly (ISUC)	23.5	0.488 ± 0.013	5.37	2.52	1997	–
9. NDVI increased significantly and NPP increased significantly (ISIS)	12.2	0.326 ± 0.010	5.66	5.55	1995	1995

**Table 2:** The correlation between the standardized anomalies (SA) of annual mean NDVI and potential NPP in different trend regions.

Different trend regions	*R*^*2*^	Climate change contribution (%)
1. NDVI decreased significantly and NPP decreased significantly (DSDS)	0.5151**	51.5
2. NDVI decreased significantly and NPP unchanged significantly (DSUC)	0.0316	3.2
3. NDVI decreased significantly and NPP increased significantly (DSIS)	0.3222**	32.2
4. NDVI unchanged significantly and NPP decreased significantly (UCDS)	0.0493	4.9
5. NDVI unchanged significantly and NPP unchanged significantly (UCUC)	0.1517*	15.2
6. NDVI unchanged significantly and NPP increased significantly (UCIS)	0.1810*	18.1
7. NDVI increased significantly and NPP decreased significantly (ISDS)	0.4092**	40.9
8. NDVI increased significantly and NPP unchanged significantly (ISUC)	0.2054*	20.5
9. NDVI increased significantly and NPP increased significantly (ISIS)	0.7120**	71.2
