# Supplementary material for: Climatic change controls productivity variation in global grasslands
Source: Sci Rep. 2016 May 31;6:26958. doi: 10.1038/srep26958 (PMC4886642; doi:10.1038/srep26958)
Supplement: Supplementary Information [file srep26958-s1.doc]

***Climatic change controls productivity variation in global grasslands***

*Qingzhu Gao*^A,B,E^, *Wenquan Zhu*^C^*, Mark W. Schwartz*^D^*, H. Ganjurjav*^A,B^*, Yunfan Wan*^A,B^, *Xiaobo Qin*^A,B^, *Xin Ma*^A,B^*, Matthew A. Williamson*^D^, *Yue Li*^A,B^

^A^ Institute of Environment and Sustainable Development in Agriculture, Chinese Academy of Agricultural Sciences, Beijing 100081, China.

^B^ Key Laboratory for Agro-Environment & Climate Change, Ministry of Agriculture, Beijing 100081, China.

^C^ College of Resources Science and Technology, Beijing Normal University, Beijing 100875, China.

^D^ John Muir Institute of the Environment, University of California, Davis, CA 95616, USA

^E^ Corresponding author. Email: [gaoqingzhu@caas.cn](mailto:gaoqingzhu@caas.cn)

**Supplementary Materials**


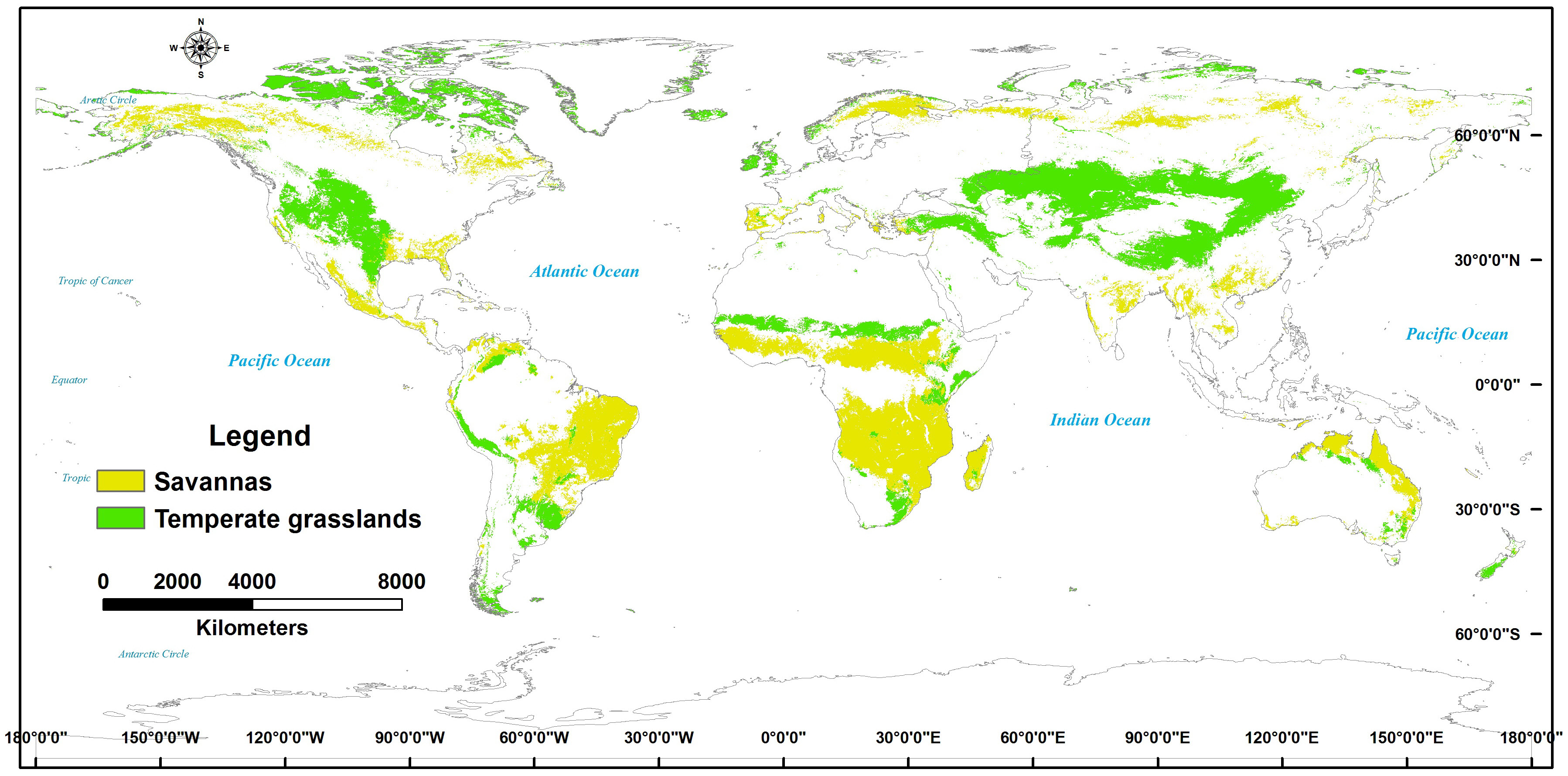


(2)

(1)

(5)

(7)

(6)

(4)

(3)

(8)

**Supplementary Figure S1 Global grassland distribution and regional division map.**

Eight major distribution regions selected for future analysis are outlined, there are (1) is the high-latitude Region, (2) is Midwest USA, (3) is Mideast South America, (4) is Central Africa, (5) is Central Eurasia, (6) is Mongolian Plateau, (7) is Qinghai-Tibetan Plateau, and (8) is Oceania

The spatial map of distribution and regional division in global grassland ecosystems was developed from the spatial overlap technique through the application of ArcGIS 10 (http://www.esri.com/software/arcgis/arcgis-for-desktop).

**Supplementary Figure S2 Trends of standardized anomalies of annual mean NDVI and climatic potential NPP in mainly distribution regions of global grassland ecosystem.**

Eight major distribution regions selected for future analysis are outlined, there are (1) is the high Latitude Region, (2) is Midwest USA, (3) is Mideast South America, (4) is Central Africa, (5) is Central Eurasia, (6) is Mongolian Plateau, (7) is Qinghai-Tibetan Plateau, and (8) is Oceania


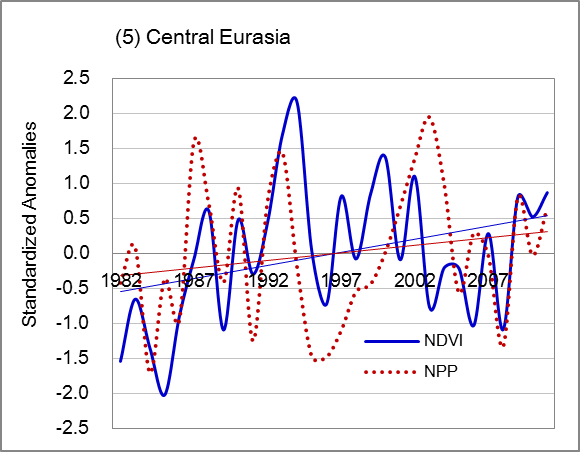

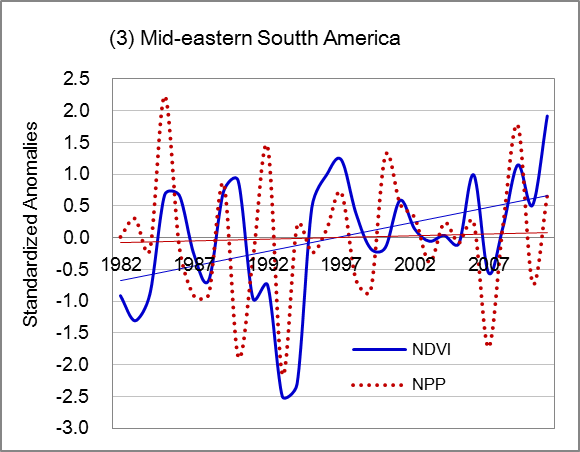

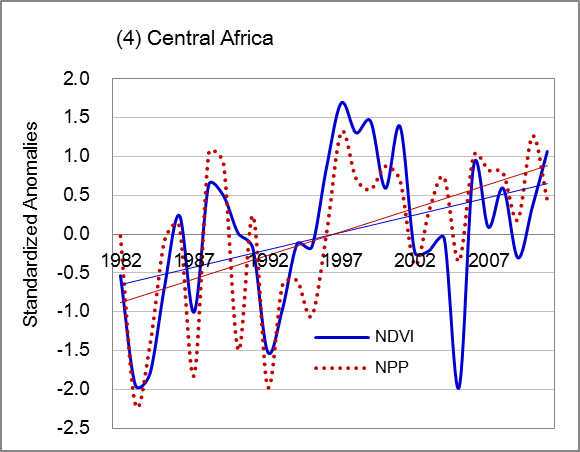

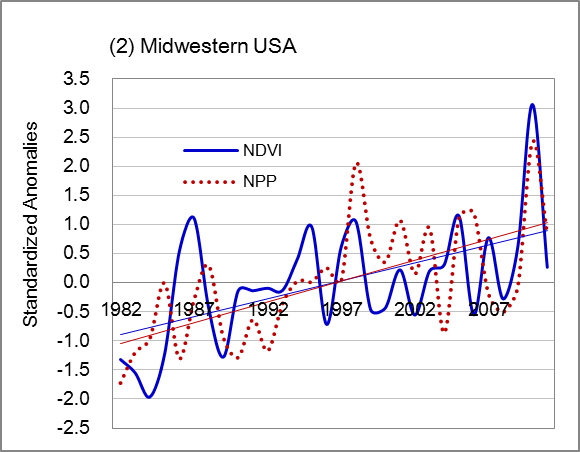

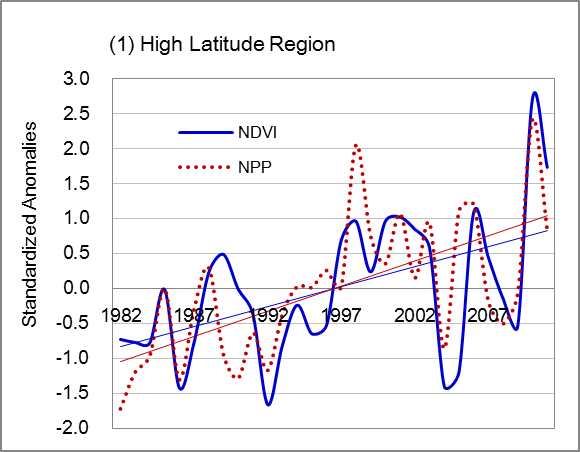

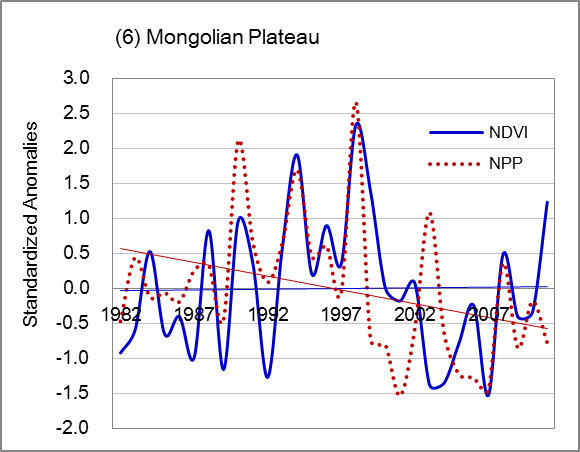

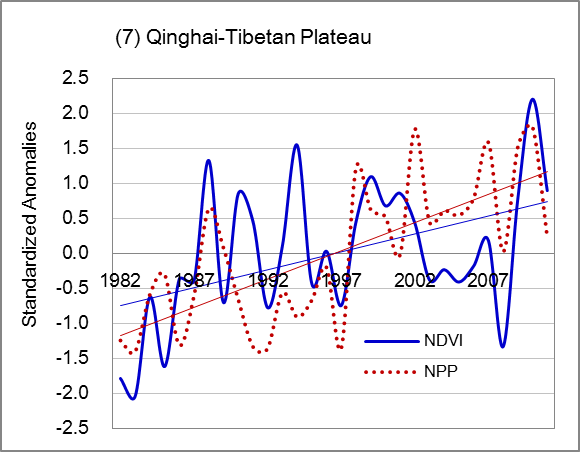

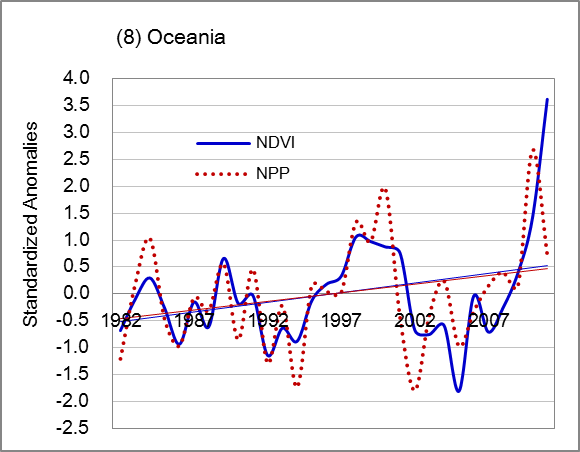


**Supplementary Table S1 the climatic conditions of major distribution regions in global grassland ecosystem**

| **Climatic Factors** | Monthly and Annual | **Major grassland distribution regions** | | | | | | | |
| --- | --- | --- | --- | --- | --- | --- | --- | --- | --- |
|  |  | The high-latitude Region | Midwest USA | Mideast South America | Central Africa | Central Eurasia | Mongolian Plateau | Qinghai-Tibetan Plateau | Oceania |
| **Precipitation**  **(mm)** | Jan. | 7.0±1.9 | 25.6±6.0 | 190.1±39.9 | 84.6±11.7 | 21.9±4.6 | 1.9±0.7 | 4.8±2.4 | 198.3±72.0 |
|  | Feb. | 6.6±1.7 | 25.2±7.5 | 175.3±26.2 | 76.2±12.4 | 20.9±5.3 | 2.1±1.0 | 7.2±3.8 | 188.8±66.5 |
|  | Mar. | 8.0±1.4 | 34.1±8.3 | 188.2±26.5 | 65.7±10.7 | 25.0±5.7 | 5.0±1.9 | 13.7±4.8 | 135.9±54.9 |
|  | Apr. | 10.1±1.9 | 39.4±9.8 | 125.4±21.2 | 49.6±8.0 | 31.0±7.6 | 11.6±3.3 | 20.2±6.3 | 58.9±31.8 |
|  | May | 12.7±2.2 | 59.1±16.3 | 65.2±11.8 | 40.0±6.4 | 35.8±6.4 | 24.9±7.9 | 37.0±8.5 | 39.0±12.6 |
|  | Jun. | 17.1±3.2 | 59.3±12.7 | 32.3±9.7 | 44.2±5.5 | 31.4±6.7 | 51.3±11.3 | 80.0±13.4 | 34.0±6.9 |
|  | Jul. | 28.5±4.0 | 45.3±9.5 | 25.4±7.2 | 67.7±7.6 | 30.3±7.3 | 84.8±21.6 | 102.6±14.2 | 31.9±9.6 |
|  | Aug. | 36.2±5.8 | 42.2±8.0 | 26.0±11.5 | 82.1±13.0 | 22.1±5.7 | 67.1±18.6 | 93.4±17.4 | 31.5±6.6 |
|  | Sep. | 28.0±4.2 | 41.9±12.3 | 48.4±12.6 | 58.7±8.8 | 17.3±5.1 | 29.1±9.4 | 60.9±9.1 | 32.3±8.9 |
|  | Oct. | 21.2±3.3 | 38.9±15.2 | 95.6±15.8 | 42.8±9.0 | 24.8±7.0 | 11.6±3.8 | 19.7±5.5 | 47.6±15.5 |
|  | Nov. | 14.6±2.7 | 28.7±10.2 | 136.9±16.7 | 45.6±8.1 | 27.2±6.6 | 5.5±1.9 | 5.6±3.5 | 74.7±23.0 |
|  | Dec. | 9.9±2.0 | 27.1±7.1 | 181.4±27.4 | 72.5±8.5 | 25.6±6.2 | 3.3±1.1 | 4.3±2.9 | 134.9±39.3 |
|  | Annual Total Precipitation | 200.0±10.6 | 466.8±43.8 | 1290.1±89.1 | 729.6±51.7 | 313.3±29.6 | 298.2±43.4 | 449.3±36.3 | 1007.8±133.5 |
| **Temperature**  **(°C)** | Jan. | -27.5±2.0 | -2.1±1.9 | 24.7±0.4 | 24.5±0.5 | -11.1±2.3 | -20.6±2.2 | -12.2±1.1 | 26.3±0.6 |
|  | Feb. | -28.1±2.2 | 0.0±2.2 | 24.6±0.4 | 25.6±0.7 | -9.9±2.7 | -16.2±2.7 | -9.9±1.5 | 25.9±0.6 |
|  | Mar. | -25.4±1.8 | 4.4±1.5 | 24.2±0.4 | 26.5±0.4 | -3.1±2.6 | -7.8±2.4 | -5.9±0.9 | 24.9±0.5 |
|  | Apr. | -17.9±1.9 | 9.2±1.2 | 23.3±0.4 | 26.5±0.5 | 7.6±1.6 | 2.8±1.7 | -1.3±0.9 | 22.9±0.5 |
|  | May | -7.8±1.5 | 14.4±0.9 | 21.9±0.4 | 25.2±0.4 | 14.4±1.0 | 10.5±1.0 | 3.1±0.6 | 20.1±0.7 |
|  | Jun. | 1.6±0.9 | 19.0±1.0 | 20.7±0.5 | 23.3±0.3 | 19.5±0.9 | 16.0±1.4 | 7.0±0.6 | 17.4±0.8 |
|  | Jul. | 6.0±0.6 | 22.5±1.0 | 20.5±0.6 | 22.2±0.4 | 21.8±0.8 | 18.5±1.1 | 9.0±0.6 | 16.8±0.7 |
|  | Aug. | 4.0±0.7 | 21.8±0.9 | 21.6±0.5 | 22.9±0.4 | 20.3±0.9 | 16.7±1.0 | 8.3±0.6 | 18.3±0.6 |
|  | Sep. | -1.1±0.9 | 16.8±1.1 | 22.9±0.5 | 24.5±0.4 | 14.5±0.8 | 10.2±1.1 | 5.3±0.6 | 21.3±0.6 |
|  | Oct. | -9.6±1.4 | 10.5±1.1 | 24.0±0.5 | 25.9±0.4 | 6.9±1.3 | 1.9±1.3 | -0.7±0.7 | 23.9±0.6 |
|  | Nov. | -18.3±2.2 | 3.4±1.8 | 24.3±0.4 | 25.6±0.5 | -1.9±2.1 | -9.7±2.1 | -7.1±0.8 | 25.6±0.4 |
|  | Dec. | -23.7±2.6 | -1.7±2.0 | 24.5±0.3 | 24.7±0.5 | -8.5±2.2 | -17.7±1.7 | -10.9±0.7 | 26.2±0.5 |
|  | Annual Mean Temperature | -12.3±0.9 | 9.9±0.5 | 23.1±0.3 | 24.8±0.3 | 5.9±0.7 | 0.4±0.7 | -1.3±0.5 | 22.5±0.3 |
